# Cryoballoon catheter ablation or drug therapy to delay progression of atrial fibrillation: A single-center randomized trial

**DOI:** 10.3389/fcvm.2022.1003305

**Published:** 2022-10-19

**Authors:** Jun Ding, Aijuan Cheng, Peng Li, Yingchuan Yan, Yutian Shi, Zuochen Xue, Shan Sun, Jing Xu

**Affiliations:** Department of Cardiology, Tianjin Chest Hospital, Tianjin, China

**Keywords:** atrial fibrillation, anti-arrhythmic drug, paroxysmal atrial fibrillation, persistent atrial fibrillation, cryoballoon catheter ablation, progression

## Abstract

**Background:**

Delaying atrial fibrillation (AF) progression is a key goal in cardiovascular treatment. However, numbers of previously published studies on delayed AF progression are relatively limited. The purpose of this study was to determine whether a cryoballoon catheter ablation (CA) strategy could delay AF progression compared to anti-arrhythmic drug (AAD) treatment in patients with paroxysmal AF.

**Methods:**

A total of 204 subjects were enrolled in the trial, including 102 in the cryoballoon CA group and 102 in the AAD group. Participants were followed up with for 36 months. The primary study endpoint was the first occurrence of persistent atrial tachyarrhythmia, while secondary endpoints included the event rates of persistent atrial tachyarrhythmia at 1 and 2 years. The primary safety endpoint was serious adverse events.

**Results:**

Overall, the 36-month follow-up was completed by 154 subjects (75.5%). At 3 years, documented progression from paroxysmal AF to persistent atrial tachyarrhythmia had occurred in 2 of the 102 patients assigned to undergo cryoballoon CA [2.203% (95% confidence interval (CI), 0.554–8.537)] and in 17 of the 102 patients assigned to receive AADs [20.223% (95% CI, 13.040–30.604)] [hazard ratio (HR), 0.107; 95% CI, 0.043–0.262; *P* < 0.001]. Lower rates of progression in the cryoballoon CA group compared to the AAD group were already obvious at 1 year [1.053% (95% CI, 0.149–7.238) vs. 5.284% (95% CI, 2.233–12.237)] [HR, 0.193; (95% CI, 0.039–0.956; *P* = 0.09)] and 2 years [2.203% (95% CI, 0.554–8.537) vs. 12.430% (95% CI, 7.066–21.371)] (HR, 0.169; 95% CI, 0.057–0.501, *P* < 0.001). Serious adverse events occurred in 7 of the 102 patients (6.9%) in the cryoballoon CA group and 9 of the 102 patients (8.8%) in the AAD group.

**Conclusion:**

Cryoballoon CA was superior to AAD therapy in preventing the occurrence of persistent atrial tachyarrhythmia in patients with paroxysmal AF who had not received prior rhythm control therapy. Serious adverse events were rare.

## Introduction

Atrial fibrillation (AF) is a heterogeneous disease with a variable natural history, origins, and clinical profile. The progression of AF is a frequently observable phenomenon in clinical medicine. The progression from paroxysmal to persistent AF has recently attracted increasing attention and has been shown to be associated with increased morbidity and mortality rates (1). The progression of AF leads to an increased risk of acute decompensation with heart failure, myocardial infarction, thromboembolism, and stroke (2, 3); delaying AF progression is therefore a key goal in cardiovascular treatment. However, previously published studies on delayed AF progression are relatively limited in number.

Several studies have shown that cryoballoon catheter ablation (CA) has a significant advantage over anti-arrhythmic drug (AAD) therapy in reducing the recurrence of atrial arrhythmias in patients with paroxysmal AF over a follow-up period of 1 year (4–6). However, few prospective studies to date have investigated the prevention of the progression to persistent AF, and no studies have evaluated the use of cryoballoon CA to prevent progression from paroxysmal to persistent AF. Therefore, the purpose of this study was to determine whether a cryoballoon CA strategy could better delay AF progression compared to AAD treatment according to current AF management guidelines in patients with paroxysmal AF.

## Materials and methods

### Trial design and study participants

This study was a single-center, prospective, open-label, randomized study conducted at Tianjin Chest Hospital that enrolled subjects 18–80 years old with paroxysmal AF. All study subjects had symptoms associated with paroxysmal AF and experienced ≥ 1 episodes of AF documented by Holter or 12-lead electrocardiography (ECG) in the 6 months prior to randomization. A total of 204 subjects were enrolled between January 2018 and November 2018. The intention-to-treat (ITT) cohort consisted of 102 subjects randomized to AAD therapy and 102 subjects randomized to cryoballoon CA, respectively. These subjects had no previous amiodarone exposure and no other class I or III AAD had been previously administered to them for > 2 weeks. The study exclusion criteria were previous left atrial (LA) ablation, acute coronary syndrome, LA size > 50 mm, persistent AF, left ventricular ejection fraction < 40%, intracardiac thrombi, a reversible cause of AF, and decompensated heart failure. The ethics committee of Tianjin Chest Hospital approved the study protocol, and all patients provided written informed consent.

### Cryoballoon catheter ablation

Patients who were randomly assigned to the cryoballoon CA group underwent pulmonary vein (PV) isolation (PVI) within 1 week after randomization. Briefly, the steps of the cryoballoon CA procedure were as follows: conscious sedation was completed before the cryoballoon CA procedure started. During the cryoballoon CA procedure, intravenous heparin was administered intermittently to maintain an activated clotting time of 300–350 s. A 28-mm second-generation cryoballoon (CB2) (Arctic Front Advance Cardiac Cryoablation Catheter; Medtronic, Minneapolis, MN, USA) was advanced through the steerable sheath into the LA and an over-the-wire delivery technique was adopted. Prior to cryoablation, a circular mapping catheter was placed in the PV ostium to record electrical activity at the outlet. A cryoballoon was positioned in the PV antrum, and contrast material was injected to determine the degree of obstruction. Our standard operating sequence was first to deal with the left superior PV, then the left inferior PV, then the right inferior PV, and finally the right superior PV. The Achieve catheter (Medtronic) was used during cryoballoon CA; the time-to-PVI was recorded when the PV potential had disappeared or dissociated from LA activity completely.

While cryoablating near the right-sided PVs, continuous phrenic nerve pacing with 10–20 mA of current and a 1.0–2.0-ms pulse width with a cycle length of 1,500 ms was required. The operator placed a hand on the patient’s abdomen to assess the strength of the diaphragm contraction. Cryoblation was immediately terminated when loss or reduction of the phrenic nerve pacing capture was confirmed. The cryoablation process was considered successful if PVI was achieved, as demonstrated by both exit and entrance conduction blocking between the LA and PV. When PV reconnection was detected, cryoenergy application of the reconnected PV was completed. To avoid confounding of the data, amiodarone was excluded from the cryoballoon CA group because of its longer half-life, and other class I or III AADs were used for ≤ 8 weeks. Subjects were maintained on anticoagulation therapy for ≥ 3 months. As the treatment strategy for a recurrent arrhythmia occurring ≥ 90 days post-ablation, i.e., the “blanking period,” the investigator’s routine clinical protocol was followed as much as possible. For these patients, if the recurrence was of a sufficient clinical severity, the addition of AAD therapy during the trial was permitted according to current AF management guidelines (7).

### Anti-arrhythmic drug therapy

Subjects randomized to AAD treatment were taking an approved drug for the treatment of AF and receiving a class I or class III AAD according to current guidelines. The selection of AAD was at the discretion of the investigator, and its dosage was also based on current guidelines. The management of recurrent arrhythmias should follow, as far as possible, the investigator’s routine clinical protocol. Every effort to prevent patients from crossing over from their assigned strategy until the primary endpoint event was made. Changes in the drug, schedule, and prescribed dose were permitted in the AAD therapy group during the study according to guidelines for the management of AF to optimize AAD therapy and avoid drug inefficacy. The use of calcium channel blockers, β-blockers, and oral anticoagulants was not bound by the study protocol.

### Study endpoint

The primary endpoint of the study was the first occurrence of persistent atrial tachyarrhythmia (AF, atrial tachycardia, or atrial flutter), following a 90-day blanking period after the initiation of the cryoballoon CA procedure or an AAD. The main hypothesis of this study was that cryoballoon CA is superior to AAD (class IC or III) as first-line treatment for a cohort of patients with no formal therapy (i.e., without prior AF catheter ablation or long-term use of AAD) presenting with symptomatic paroxysmal AF. Repeat ablation was not allowed during the trial period. The definition of persistent atrial tachyarrhythmia for this study was based on contemporary clinical guidelines and included a state of continuous atrial tachyarrhythmia that persisted for > 7 days. Whenever subjects experienced arrhythmic symptoms for > 1 day, they returned to the hospital for follow-up. Once atrial tachyarrhythmia was identified, 7-day Holter monitoring was initiated for 7 consecutive days. Persistent atrial tachyarrhythmia was identified by episodes of atrial tachyarrhythmia lasting > 7 days monitored through 7-day Holter or requiring termination by electrical cardioversion after 48 h. The primary endpoint of this study was evaluated for up to 3 years.

Secondary endpoints included event rates of the progression from paroxysmal AF to persistent atrial tachyarrhythmia at 1 and 2 years, as well as time to recurrent atrial tachyarrhythmia.

The primary safety endpoint was serious adverse events. Adverse events were defined as any undesired medical events that occurred in clinical study subjects, while a serious adverse event was defined as an adverse event that led to permanent impairment of a body function or a body structure; that led to a serious deterioration in health resulting in a life-threatening injury or illness, inpatient hospitalization, or prolonged hospitalization for > 1 day; the requirement of a surgical or medical intervention to permanent impairment to a body structure or a body function or to prevent a life-threatening illness or injury; or that led to death.

### Follow-up

The patients in the cryoballoon CA and AAD groups were scheduled for follow-up at baseline and 1, 3, 6, 12, 24, and 36 months, which included 12-lead ECGs, 24-h holter and recording of any patient symptoms. Seven-day Holter was performed if the subject’s ECG identified atrial tachyarrhythmia. The follow-ups were completed in the arrhythmia clinic of Tianjin Chest Hospital. Furthermore, subjects were interviewed by way of telephone calls outside of scheduled study visits throughout the follow-up period. Patients with any symptoms of suspected atrial tachyarrhythmia were considered eligible for ECG confirmation. At follow-ups, relevant patient data, such as the recurrence of AF, medication use, thromboembolic events, cardiovascular readmission, and reasons for cardiovascular rehospitalization or symptoms, were collected.

### Statistical analysis

In the calculations of sample size, we assumed that 3% of the patients in the cryoballoon CA group and 20% of the patients in the AAD group would progress from paroxysmal AF to persistent atrial tachyarrhythmia during the study period. On the basis of these assumptions and an expected 10% withdrawal rate, a sample size of 204 patients was calculated to be sufficient to provide ≥ 90% power for the analysis of the primary efficacy. The sample size calculation was performed with the PASS software (version 11; NCSS, Kaysville, UT).

The primary endpoint of the first occurrence of persistent atrial tachyarrhythmia was assessed using Kaplan–Meier curves and the log-rank test. Hazard ratios (HRs) and 95% confidence intervals (CIs) for the progression from paroxysmal AF to persistent atrial tachyarrhythmia were estimated using Cox proportional hazards models with no adjustment for relevant variables. Similarly, the HRs and 95% CIs of the progression from paroxysmal AF to persistent atrial tachyarrhythmia across each subgroup were evaluated using Cox proportional hazards models with adjustment for relevant variables. Adjustment variables included age, sex, duration of AF, LA diameter at baseline, previous coronary artery disease, previous heart failure, previous hypertension, previous diabetes, previous stroke or transient ischemic attack, and previous sleep apnea. The interaction of each subgroup and treatment approach was investigated by Cox proportional hazards modeling. The standard error for each percentage of patients with an event within 12, 24, and 36 months was approximated with Greenwood’s formula, and 2-sided 95% log–log confidence intervals were constructed.

The analysis of the primary endpoint was based on the ITT principle. A sensitivity analysis of the primary endpoint was performed involving the per-protocol (PP) population, which included all patients who underwent treatment according to randomization.

Variables representing a normal distribution were expressed as mean ± standard deviation (SD) values and compared using Student’s *t*-test. Categorical data were compared using the chi-squared test or Fisher’s exact test.

All reported *P*-values were calculated using a 2-tailed test, and *P* < 0.05 was considered statistically significant. All statistical analyses were conducted with the SPSS statistical software (version 19.0; IBM Corporation, Armonk, NY, USA).

## Results

### Patients

**FIGURE 1:**
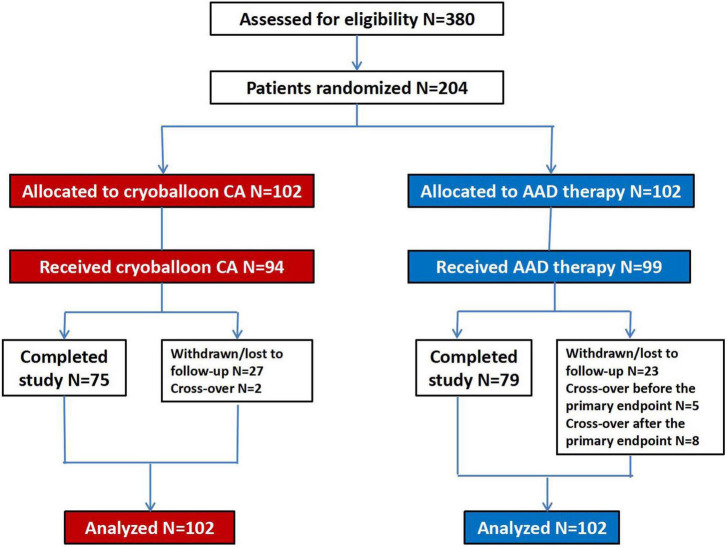
Patient flow diagram. CA, catheter ablation; AAD, anti-arrhythmic drug.

We enrolled 204 patients from January 2018 through November 2018 who received either cryoballoon CA (102 patients) or AADs (102 patients) and were included in the ITT population. Overall, the 36-month follow-up was completed by 154 subjects (75.5%). The baseline demographic data and clinical characteristics were not statistically different between the 2 groups (Table 1). Meanwhile, the PP population included 193 patients (94 with cryoballoon CA and 99 with AADs) (Figure 1).

**TABLE 1:** Baseline characteristics of the patients (ITT population).

Characteristic	Cryoballoon CA group (*n* = 102)	AAD group (*n* = 102)	*P*
Age (years)	60.90 ± 7.89	60.74 ± 10.16	0.896
Sex (female)	41 (40.20%)	42 (41.18%)	0.887
BMI (kg/m^2^)	25.74 ± 3.11	25.73 ± 3.45	0.975
Hypertension	54 (52.94%)	47 (46.08%)	0.327
Diabetes mellitus	16 (15.69%)	15 (14.71%)	0.845
Stroke/TIA	8 (7.84%)	10 (9.80%)	0.622
Coronary heart disease	25 (24.51%)	27 (26.47%)	0.748
Congestive heart failure	7 (6.86%)	10 (9.80%)	0.447
Sleep apnea	8 (7.84%)	8 (7.84%)	1.000
AF duration (m)	33.05 ± 23.95	33.07 ± 24.77	0.995
CHA_2_DS_2_-VASc score	1.65 ± 1.38	1.78 ± 1.30	0.465
LA diameter (mm)	38.29 ± 3.68	39.11 ± 3.89	0.126
LVEF (%)	60.91 ± 4.71	59.96 ± 5.00	0.164

Values are given as mean ± *SD* or *n* (%). There are no different characteristics between the groups. BMI, body mass index; CHA_2_DS_2_-VASc, score for AF and stroke risk; LVEF, left ventricular ejection fraction; TIA, transient ischemic attack.

### Treatment characteristics

In the cryoballoon CA group, complete isolation of the PVs was achieved in all 94 patients who underwent the procedure. The mean (± *SD*) duration of the procedure was 99.89 ± 12.91 min, the mean LA dwell time was 73.86 ± 10.15 min, and the mean fluoroscopy time was 12.83 ± 2.70 min. Information on drugs and dosing for the 31 subjects in the cryoballoon CA group and the 99 subjects in the AAD group are presented in Table 2 and Supplementary Tables 1, 2.

**TABLE 2:** AAD therapy and dosing in the AAD group.

Drug and daily dose	At end of blanking period (*n* = 99)	At treatment failure, 36 months, or exit (*n* = 99)
	No. of patients (%)	
Propafenone	52 (52.5%)	44 (44.4%)
300 mg	9	15
450 mg	41	22
600 mg	2	7
Dronedarone	0	11 (11.1%)
800 mg	0	11
Sotalol	31 (31.3%)	23 (23.2%)
80 mg	12	7
160 mg	16	12
240 mg	3	4
Amiodarone	8 (8.1%)	9 (9.1%)
200 mg	6	9
400 mg	2	0
Not taking a class I or III AAD	8 (8.1%)	12 (12.1%)

Values are given as *n* (%). AAD, anti-arrhythmic drug.

### Crossovers

In this study, crossover between treatment groups was allowed but rarely occurred. Crossovers occurred in 7 subjects before the occurrence of a primary endpoint event. Two subjects in the cryoballoon CA group elected not to undergo a cryoablation procedure and instead were treated with AADs. Additionally, 5 subjects in the AAD group had crossover events during the study and elected to receive cryoballoon CA. However, none of the patients in the AAD group underwent cryoballoon CA during the blanking period. In addition, 8 subjects crossed over from their assigned group after the occurrence of a primary endpoint event.

### Endpoints

**FIGURE 2:**
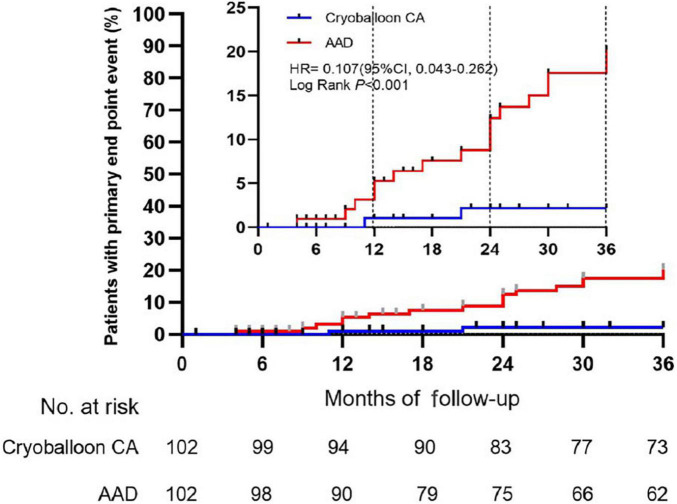
Time to the occurrence of persistent atrial tachyarrhythmia in the intention-to-treat cohort. CA, catheter ablation; AAD, anti-arrhythmic drug.

At 3 years, a documented progression from paroxysmal AF to persistent atrial tachyarrhythmia had occurred in 2 of the 102 patients assigned to undergo cryoballoon CA (2.203% [95% CI, 0.554–8.537]) and 17 of the 102 patients assigned to receive AADs (20.223% [95% CI, 13.040–30.604]) (HR, 0.107; 95% CI, 0.043–0.262; *P* < 0.001) (Figure 2, Table 3 and Supplementary Figure 1).

**TABLE 3:** Primary endpoint events within 36 months (ITT population).

Primary endpoint event	Cryoballoon CA (*n* = 102)	AAD (*n* = 102)
	No. of patients	
Persistent atrial tachyarrhythmia	2	17
persistent AF	1	12
persistent AT	1	1
persistent AFL	0	0
persistent AF, AT	0	1
persistent AF, AFL	0	2
persistent AF, AT, AFL	0	1

CA, catheter ablation; AAD, anti-arrhythmic drug; AF, atrial fibrillation; AT, atrial tachycardia; AFL, atrial flutter.

Lower rates of progression (secondary endpoint) in the cryoballoon CA group compared to the AAD group were already obvious at 1 and 2 years as follows: 1.053% (95% CI, 0.149–7.238) vs. 5.284% (95% CI, 2.233–12.237) (HR, 0.193; 95% CI, 0.039–0.956; *P* = 0.09) at 1 year and 2.203% (95% CI, 0.554–8.537) vs. 12.430% (95% CI, 7.066–21.371) (HR, 0.169; 95% CI, 0.057–0.501; *P* < 0.001) at 2 years, respectively. Over the 3-year follow-up period, The incidence of recurrent atrial tachyarrhythmia and recurrent AF was lower in the cryoballoon CA group than the AAD group (Supplementary Figures 2, 3).

### Sensitivity analyses

**FIGURE 3:**
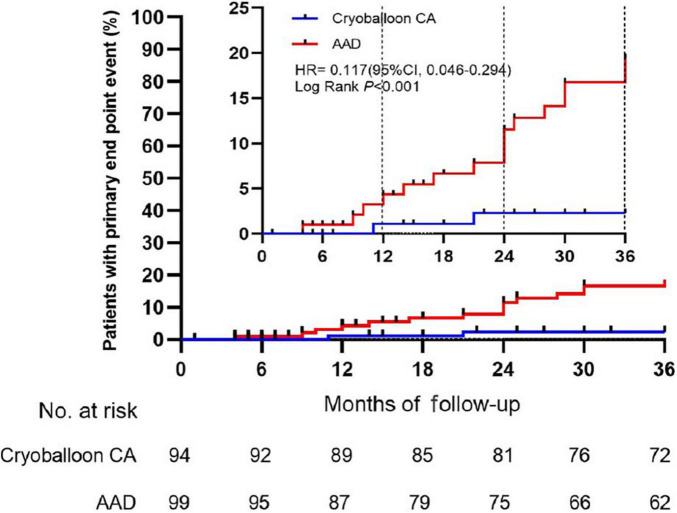
Time to the occurrence of persistent atrial tachyarrhythmia in the per-protocol cohort. CA, catheter ablation; AAD, anti-arrhythmic drug.

**FIGURE 4:**
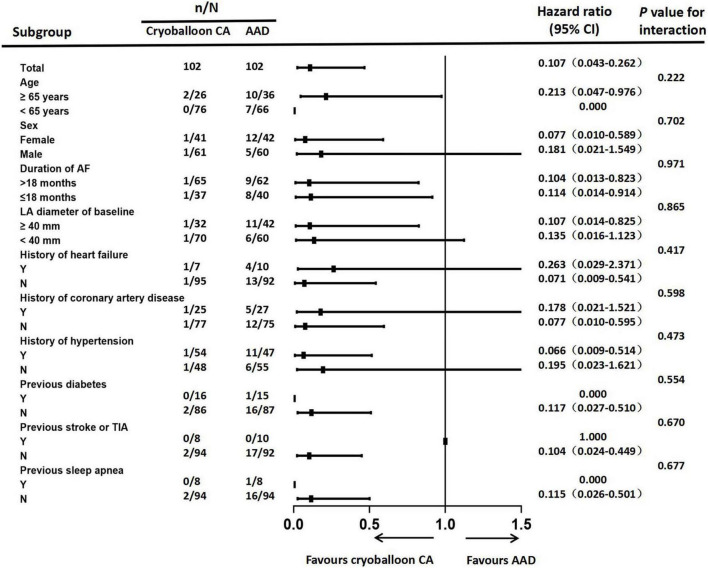
Forest plot of progression from paroxysmal atrial fibrillation to persistent atrial tachyarrhythmia. Subgroup analyses of atrial fibrillation progression were performed according to patients’ baseline conditions. CA, catheter ablation; AAD, anti-arrhythmic drug; AF, atrial fibrillation; LA, left atrial; TIA, transient ischemic attack.

Sensitivity analyses of the primary endpoint in the PP population confirmed these results. In the PP analysis, the 3-year Kaplan–Meier estimate of the rate of persistent atrial tachyarrhythmia was significantly lower in the cryoballoon CA group than the AAD group (HR, 0.117; 95% CI, 0.046–0.294; *P* < 0.001) (Figure 3). In the ITT population, there were no interactions between baseline conditions and treatment assignment according to the occurrence of AF progression from paroxysmal AF to persistent atrial tachyarrhythmia (Figure 4).

### Safety outcomes

All adverse events are listed in Table 3. Serious adverse events occurred in 7 of the 102 patients (6.9%) in the cryoballoon CA group and 9 of the 102 patients (8.8%) in the AAD group. These events included 2 cases of vascular pseudoaneurysm, 2 cases of phrenic nerve palsy, 2 cases of symptomatic bradycardia, and 1 case of hospitalization for recurrent atrial arrhythmia in the cryoballoon CA group as well as 1 case of acute coronary syndrome, 4 cases of hospitalization for recurrent atrial arrhythmia, and 4 cases of symptomatic bradycardia in the AAD group (Table 4).

**TABLE 4:** Adverse events.

Event, *n* (%)	Cryoballoon CA group (n = 102)	AAD group (n = 102)
**Any serious adverse event**		
Patients (%)	7 (6.9%)	9 (8.8%)
Events	7	9
**Any adverse event**		
Patients (%)	14 (13.7%)	13 (12.7%)
Events	16	17
Death	0	0
**Cardiac event**		
Pericardial effusion	1	0
Bradycardia	2	4
Tachycardia	3	1
Acute coronary syndrome	0	1
**Pulmonary event**		
Phrenic nerve palsy	2	0
Lung disorder	1	0
**Cerebrovascular event**		
Stroke	0	0
Transient ischemic attack	1	1
**Vascular event**		
Hematoma	1	0
Vascular pseudoaneurysm	2	0
**Gastrointestinal event**		
Atrio-esophageal fistula	0	0
Impaired gastric emptying	1	0
Major bleeding events	0	0
Bleeding events	1	3
Adverse drug reaction	0	3
Hospitalization for recurrent atrial arrhythmia	1	4

Values are given as *n* (%). CA, catheter ablation; AAD, anti-arrhythmic drug.

## Discussion

In the present study involving patients with paroxysmal AF without previous regular AAD treatment, we found that the progression from paroxysmal to persistent AF significantly occurred less often with use of an initial strategy of cryoballoon CA compared to one of AAD therapy at 3 years. So far, according to relevant guidelines, cryoballoon CA of PVI is recommended mainly as second-line treatment for symptomatic paroxysmal AF after ≥ 1 AAD treatment is not satisfactory. Several recent randomized controlled studies have demonstrated that initial treatment with cryoballoon CA is more effective than AAD therapy for preventing atrial tachyarrhythmia recurrence in patients with naive symptomatic paroxysmal AF. The STOP AF FIRST study concluded that cryoballoon CA as an initial therapy was superior to medical therapy in preventing recurrence of atrial arrhythmias in patients with paroxysmal AF (4). The EARLY-AF study also inferred the same conclusions through continuous cardiac rhythm monitoring (5). The Cryo-FIRST trial showed that first-line cryoballoon CA was associated with a higher rate of symptom resolution and obvious improvements in AF-specific quality of life compared to AAD treatment (8). Our study provides new evidence supporting the use of cryoballoon CA as an initial first-line treatment.

Our results strongly support the increased benefit of cryoballoon CA. This study, to our knowledge, is the first randomized trial to use the progression of paroxysmal to persistent AF as a clinical endpoint in a cryoballoon CA AF trial. Some of the previous AF studies have shown that progression from paroxysmal to persistent AF occurred at rates of 4.9–8.6% at 1 year (9–12). At 10 years after the first onset of paroxysmal AF, > 50% of the patients had died or progressed to persistent AF, including 36.3% who had progressed to persistent AF. Although previous studies have debated whether rhythm-control interventions reduce AF progression, most studies have demonstrated that PVI is associated with lower rates of AF progression. However, in these studies, PVI was achieved by radiofrequency ablation (13–16), and none considered cryoballoon CA as a therapy. In the ATTEST study, the rate of progression to persistent AF in patients with paroxysmal AF treated with radiofrequency catheter ablation was 1.3% at 1 year and 2.4% at 3 years (17). To our knowledge, the annual rate of progression to persistent AF in patients with paroxysmal AF treated with cryoballoon CA has not yet been reported.

The progression of paroxysmal to persistent AF involves structural and electrophysiological changes or abnormalities. These changes may involve age-dependent conduction disorders, atrial dilation, increased LA stiffness, increased myocardial fibrosis, or electroanatomic changes. Several previous studies have confirmed that increasing age, hypertension, heart failure, LA dilation, duration of AF, and other factors are associated with progression from paroxysmal to persistent AF (18–22). Given the progressive pattern over time, AF may be recognized as a dynamical disease characterized by abnormal temporal organization. Previous studies have suggested that trigger activity of PVs and autonomic nervous system regulation interact with an abnormal atrial substrate to promote progression from paroxysmal to permanent AF (23, 24). The means by which cryoballoon CA delays the progression of paroxysmal to persistent AF may involve the following mechanisms. First, cryoballoon CA is able to effectively eliminate trigger activity of the PVs, which may be the main mechanism by which cryoablation delays the progression of paroxysmal to persistent AF. Second, however, previous studies have provided evidence that AF itself is a key pathogenic factor in the progression of atrial remodeling in humans (25); notably, paroxysmal AF progression is associated with AF burden. Therefore, cryoballoon CA could play a key role in the prevention of progression from paroxysmal to persistent AF by reducing AF burden. Third, parasympathetic denervation caused by cryoballoon CA may prevent AF progression.

Our patients with AF exhibited significant clinical heterogeneity, and we performed subgroup analyses to exclude the influence of confounding factors on the conclusions of this study. In our investigation, none of the baseline conditions showed a significant interaction with cryoballoon ablation or AAD therapy in delaying the progression from paroxysmal AF to persistent atrial tachyarrhythmia. The subgroup analysis was powered to support overall comparisons.

Safety is very important to consider with first-line use of cryoballoon CA in the treatment of paroxysmal AF. Since both treatment strategies are associated with different types of adverse events, the safety of cryoballoon CA and AADs cannot be compared in a meaningful way. Serious complications associated with cryoballoon CA were uncommon in the present study. There were no occurrences of death, major bleeding events, pericardial tamponade, stroke, or atrio-esophageal fistula within the cryoballoon CA group. Overall, these findings are consistent with those recently reported in EARLY-AF, Cryo-FIRST, and STOP AF FIRST; all of these studies observed that serious adverse events associated with cryoballoon CA were infrequent. Furthermore, these results are supported by the extensive literature that cryoballoon CA can be performed safely by skilled operators.

### Limitations

Several limitations of our study should be acknowledged. First, this was a single-center trial and neither patients nor clinicians were blinded, which may have introduced bias and reduced generalizability in the study process. Second, although we have standardized follow-up procedures, the rate of patient loss to follow-up was relatively high with longer follow-up. Third, approximately 1/3 of patients in the cryoballoon CA group started the AAD regimen after the blanking period during the study, which may have been an uncontrolled confounding factor. Fourth, the use of intermittent ECG monitoring or symptoms in our study likely underestimated the rate of AF progression. Fifth, some patients unable to tolerate higher doses of AAD had their doses reduced based on clinical judgment. Therefore, these patients in the AAD group may be undertreated, which may overestimate the benefit of Cryoballoon CA.

## Conclusion

In this randomized, single-center trial, cryoballoon CA was superior to AAD therapy in preventing the occurrence of persistent atrial tachyarrhythmia in patients with paroxysmal AF with no prior rhythm control therapy. Serious adverse events were rare.

## Data availability statement

The raw data supporting the conclusions of this article will be made available by the authors, without undue reservation.

## Ethics statement

The studies involving human participants were reviewed and approved by the Ethics Committee of Tianjin Chest Hospital. The patients/participants provided their written informed consent to participate in this study.

## Author contributions

JD, SS, and JX contributed to the conception or design of the work. JD, AC, PL, YY, YS, and ZX contributed to the acquisition, analysis, or interpretation of data. JD drafted the manuscript. JX and SS critically revised the manuscript. All authors read and approved the final manuscript.
