# Supplementary material for: Cryoballoon catheter ablation or drug therapy to delay progression of atrial fibrillation: A single-center randomized trial
Source: Front Cardiovasc Med. 2022 Oct 19;9:1003305. doi: 10.3389/fcvm.2022.1003305 (PMC9627306; doi:10.3389/fcvm.2022.1003305)
Supplement: Supplementary file 1 [file Data_Sheet_1.docx]

**Table S1. AAD therapy and dosing in the cryoballoon CA group.**

| **Drug and Daily Dose** | **At the Beginning of AAD Treatment (n=31)** | **At Treatment Failure, 36 Months, or Exit (n=31)** |
| --- | --- | --- |
|  | **No. of patients (%)** | |
| **Propafenone** | 15 (48.4%) | 13 (42.0%) |
| **300 mg** | 2 | 5 |
| **450 mg** | 13 | 8 |
| **600 mg** | 0 | 0 |
| **Dronedarone** | 5 (16.1%) | 4 (12.9%) |
| **800 mg** | 5 | 4 |
| **Sotalol** | 9 (29.0%) | 11 (35.5%) |
| **80 mg** | 3 | 4 |
| **160 mg** | 6 | 4 |
| **240 mg** | 0 | 3 |
| **Amiodarone** | 2 (6.5%) | 3 (9.7%) |
| **200 mg** | 0 | 3 |
| **400 mg** | 0 | 0 |
| **600 mg** | 2 | 0 |

Values are given as n (%). *Abbreviation:* CA, catheter ablation; AAD, anti-arrhythmic drug.

**Table S2. All AAD therapy and dosing during study period.**

| **Drug and Daily Dose** | **Cryoballoon CA Group (n=9)** | **AAD Group**  **(n=90)** |
| --- | --- | --- |
| **6-month follow-up** | **No. of patients (%)** | |
| **Propafenone** | 6 (66.7%) | 51 (56.7%) |
| **300 mg** | 1 | 12 |
| **450 mg** | 5 | 33 |
| **600 mg** | 0 | 6 |
| **Dronedarone** | 0 | 0 |
| **800 mg** | 0 | 0 |
| **Sotalol** | 3 (33.3%) | 33 (36.7%) |
| **80 mg** | 1 | 7 |
| **160 mg** | 2 | 20 |
| **240 mg** | 0 | 6 |
| **Amiodarone** | 0 | 6 (6.7%) |
| **200 mg** | 0 | 6 |
| **400 mg** | 0 | 0 |
| **600 mg** | 0 | 0 |

| **Drug and Daily Dose** | **Cryoballoon CA Group (n=23)** | **AAD Group**  **(n=79)** |
| --- | --- | --- |
| **12-month follow-up** | **No. of patients (%)** | |
| **Propafenone** | 12 (52.2%) | 41 (51.9%) |
| **300 mg** | 1 | 13 |
| **450 mg** | 7 | 19 |
| **600 mg** | 4 | 9 |
| **Dronedarone** | 0 | 0 |
| **800 mg** | 0 | 0 |
| **Sotalol** | 9 (39.1%) | 29 (36.7%) |
| **80 mg** | 1 | 4 |
| **160 mg** | 5 | 18 |
| **240 mg** | 3 | 7 |
| **Amiodarone** | 2 (8.7%) | 9 (11.4%) |
| **200 mg** | 2 | 9 |
| **400 mg** | 0 | 0 |
| **600 mg** | 0 | 0 |

| **Drug and Daily Dose** | **Cryoballoon CA Group (n=24)** | **AAD Group**  **(n=63)** |
| --- | --- | --- |
| **24-month follow-up** | **No. of patients (%)** | |
| **Propafenone** | 11 (45.8%) | 29 (46.0%) |
| **300 mg** | 2 | 9 |
| **450 mg** | 6 | 15 |
| **600 mg** | 3 | 5 |
| **Dronedarone** | 4 (16.7%) | 9 (14.3%) |
| **800 mg** | 4 | 9 |
| **Sotalol** | 8 (33.3%) | 21 (33.3%) |
| **80 mg** | 1 | 5 |
| **160 mg** | 6 | 11 |
| **240 mg** | 1 | 5 |
| **Amiodarone** | 1 (4.2%) | 4 (6.3%) |
| **200 mg** | 1 | 4 |
| **400 mg** | 0 | 0 |
| **600 mg** | 0 | 0 |

| **Drug and Daily Dose** | **Cryoballoon CA Group (n=27)** | **AAD Group**  **(n=50)** |
| --- | --- | --- |
| **36-month follow-up** | **No. of patients (%)** | |
| **Propafenone** | 13 (48.1%) | 23 (46.0%) |
| **300 mg** | 3 | 6 |
| **450 mg** | 7 | 13 |
| **600 mg** | 3 | 4 |
| **Dronedarone** | 4 (14.8%) | 8 (16.0%) |
| **800 mg** | 4 | 8 |
| **Sotalol** | 8 (29.6%) | 16 (32.0%) |
| **80 mg** | 2 | 4 |
| **160 mg** | 5 | 9 |
| **240 mg** | 1 | 3 |
| **Amiodarone** | 2 (7.4%) | 3 (6.0%) |
| **200 mg** | 2 | 3 |
| **400 mg** | 0 | 0 |
| **600 mg** | 0 | 0 |

Values are given as n (%). *Abbreviation:* CA, catheter ablation; AAD, anti-arrhythmic drug.
