# Supplementary figures and images for: Cryoballoon catheter ablation or drug therapy to delay progression of atrial fibrillation: A single-center randomized trial
Source: Front Cardiovasc Med. 2022 Oct 19;9:1003305. doi: 10.3389/fcvm.2022.1003305 (PMC9627306; doi:10.3389/fcvm.2022.1003305)

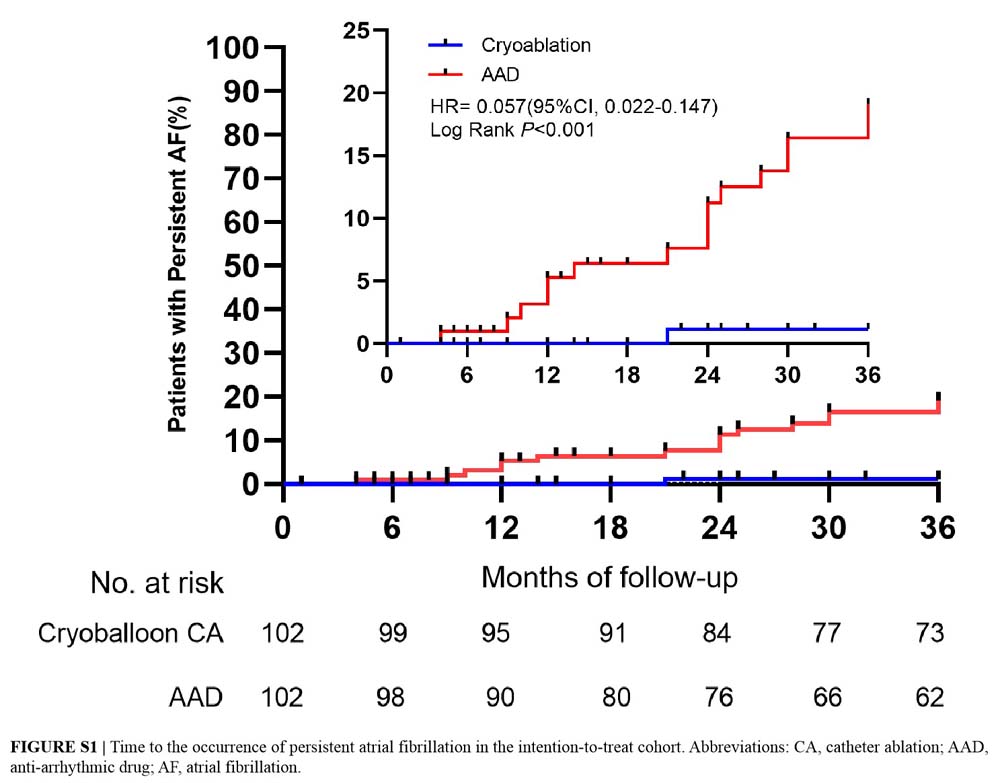

Supplement: Supplementary file 2 [file Image_1.JPEG]

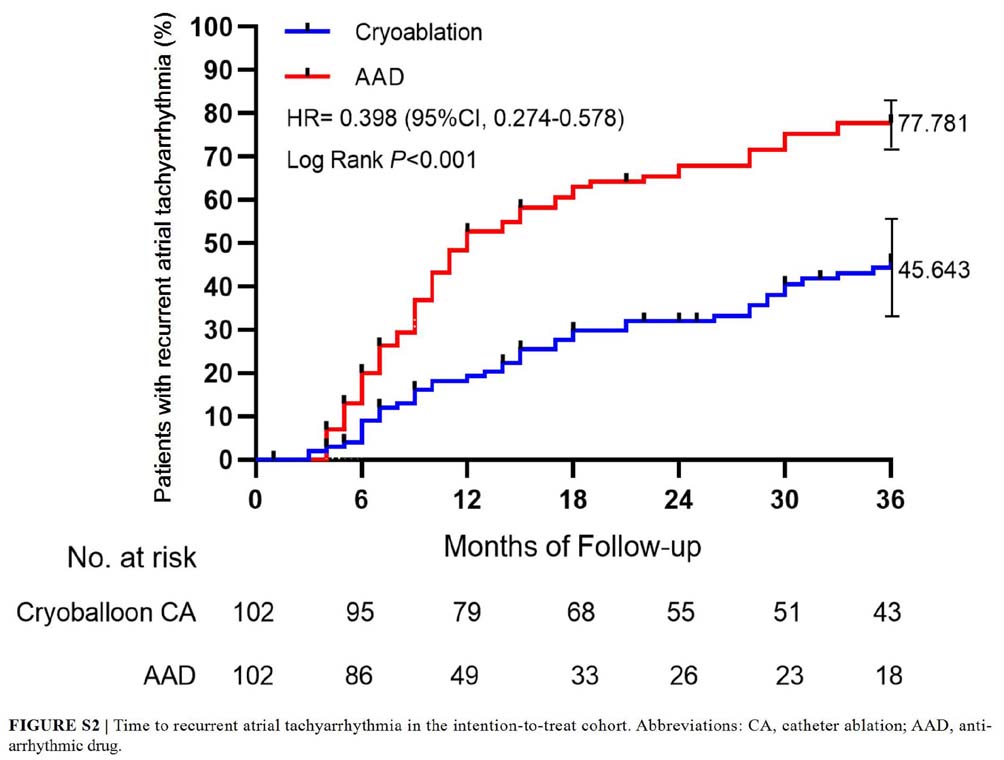

Supplement: Supplementary file 3 [file Image_2.JPEG]

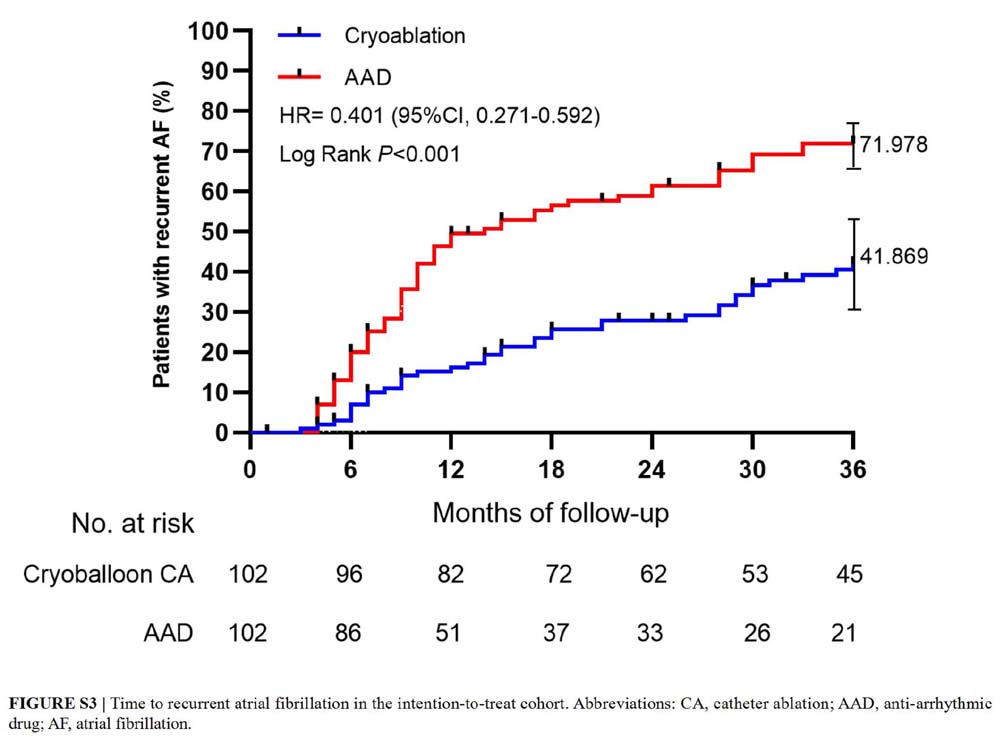

Supplement: Supplementary file 4 [file Image_3.JPEG]
